# Medulloblastoma with transitional features between Group 3 and Group 4 is associated with good prognosis

**DOI:** 10.1007/s11060-018-2797-5

**Published:** 2018-02-09

**Authors:** Maria Łastowska, Joanna Trubicka, Magdalena Niemira, Magdalena Paczkowska-Abdulsalam, Agnieszka Karkucińska-Więckowska, Magdalena Kaleta, Monika Drogosiewicz, Marta Perek-Polnik, Adam Krętowski, Bożena Cukrowska, Wiesława Grajkowska, Bożenna Dembowska-Bagińska, Ewa Matyja

**Affiliations:** 1 Department of Pathology, The Children’s Memorial Health Institute, Av. Dzieci Polskich 20, 04-730 Warsaw, Poland; 2 Clinical Research Centre, Medical University of Białystok, Skłodowskiej-Curie 24a Street, 15-276 Białystok, Poland; 3 Clinic of Oncology, The Children’s Memorial Health Institute, Av. Dzieci Polskich 20, 04-730 Warsaw, Poland; 4 Department of Endocrinology, Diabetology and Internal Medicine, Medical University of Białystok, Skłodowskiej-Curie 24a Street, 15-276 Białystok, Poland; 5 Department of Experimental and Clinical Neuropathology, Mossakowski Medical Research Centre Polish Academy of Sciences, A. Pawińskiego 5 Street, 02-106 Warsaw, Poland

**Keywords:** Medulloblastoma, Intermediate 3/4 group

## Abstract

Medulloblastoma, the most common malignant pediatric brain tumor, is a heterogeneous disease, with the existence of at least four molecular types: Wingless (WNT), Sonic Hedgehog (SHH), Group 3 and Group 4 tumors. The latter two groups, which can be identified by an application of multi-gene expression or methylation profiling, show sometimes ambiguous categorization and are still classified for diagnostic reason as non-SHH/non-WNT medulloblastomas in updated WHO 2016 classification. In order to better characterize non-SHH/non-WNT tumors, we applied the method based on the Nanostring nCounter Technology, using the 26 genes codeset in 68 uniformly treated medulloblastoma patients. This allowed for identification of tumors, which shared common Group 3 and Group 4 gene signatures. We recognized three transcriptional groups within non-WNT/non-SHH tumors: Group 3, Group 4 and the Intermediate 3/4 Group. Group 3, in line with previously published results, showed poor prognosis with survival rate < 40%, frequent metastases, large cell/anaplastic pathology and presence of tumors with *MYCC* amplification. This is in contrast to patients from the Intermediate 3/4 Group who showed the best survival rate (100%). Overall and progression free survival were better for this group than for Group 3 (p = 0.001, for both) and Group 4 (p = 0.064 and p = 0.066, respectively). Our work supports the view that within the non-WNT/non-SHH tumors different risk groups exist and that the current two groups classifier may be not sufficient for proper clinical categorization of individual patients.

## Introduction

Medulloblastoma is the most common malignant pediatric brain tumor. Recent studies revealed molecular heterogeneity of the disease, with the existence of at least four subtypes, which depend on distinctive profiles of gene expression and DNA alterations: Wingless (WNT), Sonic Hedgehog (SHH), Group 3 and Group 4 medulloblastomas [1–5].

These four molecular subtypes are now recognized as distinct biological entities and are acknowledged in the updated World Health Organization (WHO) 2016 classification of tumors of the central nervous system [6]. However, Group 3 and Group 4 tumors display several clinical and genetic overlapping features, e.g. similar location and presence of isochromosome 17q, and it is not possible currently to identify them without an application of multi-gene expression or methylation profiling. Therefore, Group 3 and Group 4 tumors, although recognized, are still classified for diagnostic reason as non-SHH/non-WNT medulloblastomas in the recent WHO 2016 classification.

In addition to microarray analysis, Group 3 and Group 4 tumors may be identified by an application of Nanostring nCounter Technology, which allows for transcriptional profiling of tumors from formalin-fixed paraffin-embedded (FFPE) tissue samples [7]. This is an important advantage, as FFPE blocks are routinely prepared in every day pathological practice. The authors proposed a 22 gene medulloblastoma classifier, which demonstrated a strong correlation with exon expression microarrays results. However, as the WNT and SHH tumors were clearly recognizable by both methods, up to 10% of tumors from Group 3 and Group 4 showed ambiguous categorization, depending on the class prediction algorithms applied.

Two recently published studies based on NanoString method and 22-genes classifier have been employed for detection of four molecular groups in medulloblastoma and the results showed that a subset of tumors displayed expression of genes from both Group 3 and Group 4 signatures. Remarkably, these tumors were classified as Group 3 in a series by Li et al. [8] but as Group 4 in our series by Łastowska et al. [9]. Since Group 3 tumors are associated with a worse prognosis [2, 4, 5] it is very important that an individual tumor is classified correctly, as this may result in clinical consequences.

Therefore, in this study, in order to better characterize non-SHH/non-WNT tumors, we applied the NanoString method using an increased 26 genes codeset, which includes four additional genes expressed in Group 3 or/and Group 4. The molecular results were correlated with histological and clinical features, including survival time of uniformly treated patients.

## Materials and methods

### Patients and tumor material

Sixty-eight patients diagnosed with medulloblastoma between the years 2003 and 2013 in The Children’s Memorial Health Institute (CMHI) in Warsaw, Poland, were included in the analysis. Only patients who were treated according to the uniform protocol of the Polish Pediatric Neurooncology Group (PPNG) for children above 3 years of age were included in survival analyses. Since patients with Group 4 tumors are characterized by later relapses our analyzed cohort had at least 4 years of observation time.

Presence of metastases at diagnosis was assessed according to Chang et al. [10].

Informed consent was obtained to use tumor material according to the procedures outlined by the CMHI’s Ethical Committee.

Analysis was performed on FFPE and frozen tumor samples obtained at diagnosis. All tumors were independently reviewed by two experienced pathologists and histologically defined according to the recent WHO 2016 morphological criteria of medulloblastoma [6].

### Detection of the molecular subtypes of tumors at the RNA level

For identification of the molecular groups NanoString nCounter system analysis (NanoString Technologies, Seattle, USA) was applied in a series of 68 medulloblastoma tumors. Total RNA was extracted from FFPE or frozen tumor samples using RNeasy kits (Qiagen). RNA integrity was assessed using an Agilent 2100 Bioanalyzer.

For group assignment a custom NanoString CodeSet was applied, which consisted of 26 marker genes and three housekeeping genes (*ACTB, GAPDH* and *LDHA*). The marker genes included 22 genes as described by Northcott et al. [7] and four additional genes: (1) *SNCAIP*, since it is duplicated and expressed in Group 4 [5], (2) *MYCC*, since it is amplified and highly expressed in some tumors from Group 3 [3], as well as two retinal differentiation genes: (3) *RCVRN* (recoverin) and (4) *PDC* (phosducin), since photoreceptor signature genes were expressed in the subset of non-WNT/non-SHH type of tumors [1, 3].

The probes were designed to target the following regions of the genes:

- for *SNCAIP* (RefSeq:NM_001242935.1), region of exon 4 and 5 TATTCATTACGCAGGTTGCTATGGCCAGGAAAAGATTCTTCTGTGGCTTCTTCAGTTTATGCAAGAACAGGGCATCTCGTTGGATGAAGTAGACCAGGAT
- for *MYCC* (RefSeq:NM_002467.3), region of exon 3 CACCGAGGAGAATGTCAAGAGGCGAACACACAACGTCTTGGAGCGCCAGAGGAGGAACGAGCTAAAACGGAGCTTTTTTGCCCTGCGTGACCAGATCCCG
- for *RCVRN* (RefSeq:NM_002903.2), region of exon 1 and 2 CCCTCTACGACGTGGACGGTAACGGGACCATCAGCAAGAATGAAGTGCTGGAGATCGTCATGGCTATTTTCAAAATGATCACTCCCGAGGACGTGAAGCT
- for *PDC* (RefSeq:NM_002597.4), region of exon 4 ACTGCCTTCGTAAATACCGTAGACAGTGTATGCAGGATATGCACCAGAAGCTGAGTTTTGGGCCTAGATATGGGTTTGTGTATGAGCTGGAAACTGGAAA,

Hybridization of the probes was performed in NanoString Technologies, Seattle, USA for an initial set of 44 tumors. An additional set of 24 tumors was analyzed in the Clinical Research Centre, Medical University of Białystok, Poland, according to NanoString Technologies procedures for hybridization, detection and scanning. Raw counts for each gene underwent technical and biological normalization using nSolver 2.5 software. Clustering of the samples was performed with Pearson, Spearman and Eucledian distance metrics and average settings.

### Detection of the PDC expression by immunohistochemistry

Expression of the *PDC* gene at the protein level (PHOS, phosducin) was detected using anti-PHOS antibody (ab77523, Abcam, dilution 1:100). Antigen retrieval was performed using Target Retrieval Solution Citrate pH 6 (DAKO), for 20 min at 99 °C. Whole preparations were scanned in Hamamatsu NanoZoomer 2.0 RS scanner at an original magnification of x40. Positive reaction was considered where areas with > 10% positive cells were encountered within examined tumor samples.

### Detection of DNA changes in the molecular subtypes of tumors

Procedures for detection of mutations in exon 3 of *CTNNB1* and detection of *MYCC* amplification by FISH are described elsewhere [11].

Detection of isochromosome 17q or 17q gain was carried out using MLPA (Multiplex Ligation-dependent Probe Amplification) analysis of genomic DNA extracted from the frozen tumor tissues. The analysis was performed using the SALSA MLPA kit P301-A2 (MRC-Holland; Amsterdam, the Netherlands) according to the manufacturer’s protocol. Probe amplification products were run on ABI Prism 3130 Genetic Analyzer (Applied Biosystems; Foster City, CA, USA). The peak plots were visualized and normalized, and the dosage ratios were calculated using GeneMarker software v 2.2.0 (Soft Genetics; LLC, State Collage, PA, USA).

### Statistical analysis

Statistical analyses were performed using T-test and Fisher Exact test. Overall survival (OS) and progression free survival (PFS) were calculated using Kaplan–Meier estimation and group comparisons were made using the log-rank test. All patients had at least 4 years of the follow-up period.

## Results

### Patients and tumors characteristics

The average age of 68 pediatric patients at diagnosis was 9.2 years, range 0.5–17 years. 43 patients were males, 25 patients were females. The tumors were histologically diagnosed as classic medulloblastoma in 48 cases, large cell/anaplastic (LCA) in 12 cases, desmoplastic/nodular (DN) in 7 cases, and not otherwise specified (NOS) in 1 case.

### Detection of molecular groups at the RNA level

An initial set of 44 tumors was analyzed by the NanoString method for assignment into one out of four molecular groups. Our set of tumors included 6 patients with mutation in exon 3 of *CTNNB1* to ensure the presence of the WNT group in the analyzed series.

**Fig. 1:**
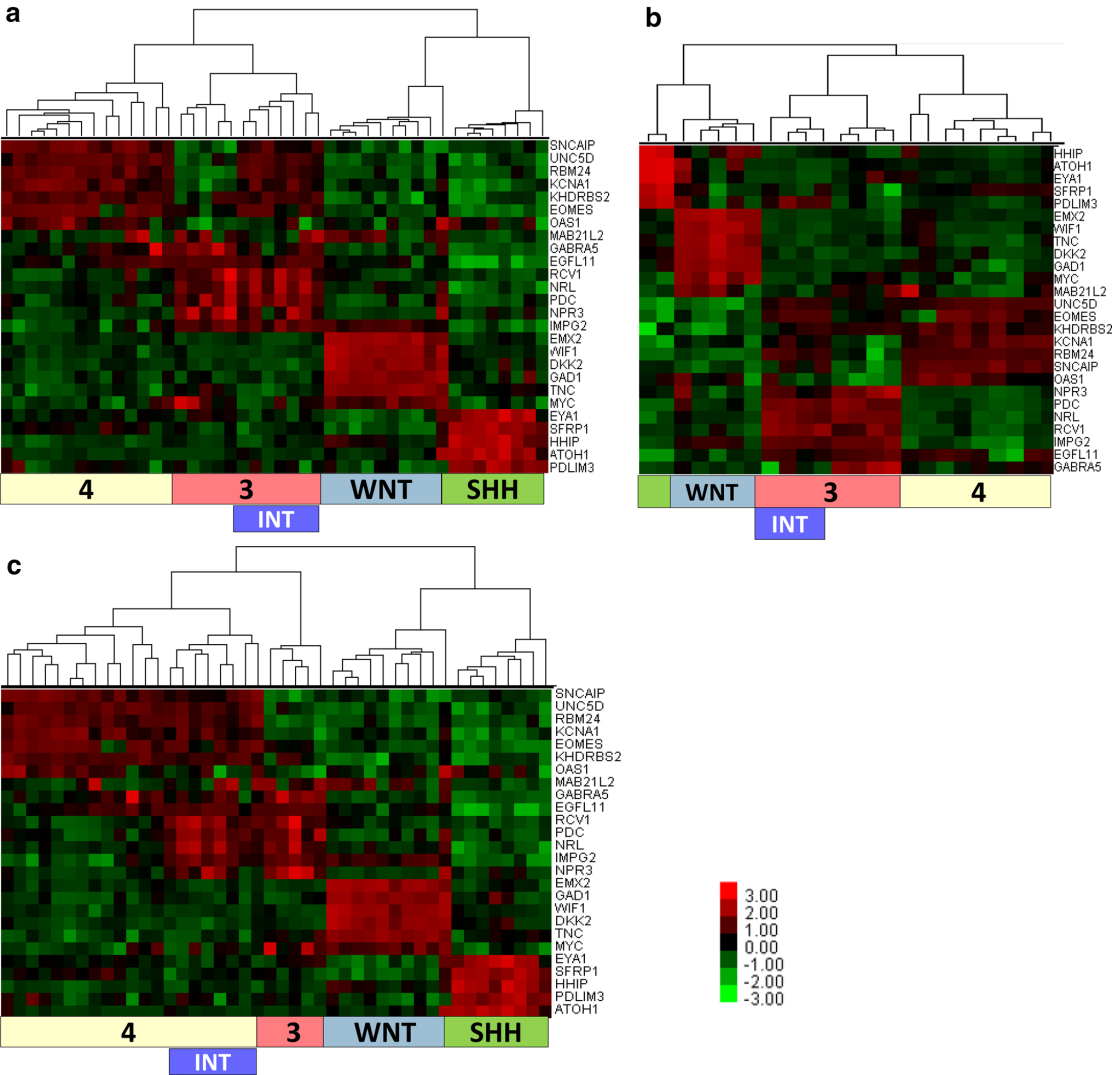
Clustering of medulloblastomas into four molecular groups. **a, b** Clustering using Pearson distance with average linkage in a series of 44 and 24 tumors. **c** Clustering of medulloblastomas into four molecular groups using Spearman distance with average linkage of the same set of 44 tumors as in part a. Colours represent log^2^ gene expression differences

Unsupervised hierarchical clustering using Pearson correlation and average settings identified four clusters of tumors: 10 tumors from the WNT subtype, 8 tumors from the SHH subtype and two clusters from the non-SHH/non-WNT subtype: cluster 3 with 12 tumors (Group 3) and cluster 4 with 14 tumors (Group 4). However, the analysis revealed that Group 3 parent cluster consisted of two sub-clusters, one with 5 tumors and the second with 7 tumors. The latter sub-cluster was characterized by expression of genes which belong both to Group 3 and Group 4 signatures. Therefore, we named this sub-cluster as the Intermediate 3/4 Group (Fig. 1a). To confirm this pattern, we performed analysis on additional 24 cases in a different laboratory (Białystok, Poland) using the same CodeSet of 26 marker genes. The raw data were analyzed using again Pearson correlation and average settings and the results matched the initial findings found in 44 tumors series. Namely, within Group 3 we could identify two sub-clusters, including four tumors, which belong to the Intermediate 3/4 Group (Fig. 1b).

### Classification of tumors to molecular groups may depend on the algorithm applied

In addition to described clustering, we performed further analyses on the same set of 44 tumors and 26 marker genes using the average linkage method with Spearman and Eucledian distance metrics, provided by nSolver 2.5 software. Both calculations identified tumors from Intermediate 3/4 Group, however, they were clustered now within Group 4 (Fig. 1c).

We assume that Intermediate 3/4 Group may represent a discreet entity as compared to “true” Group 3 or Group 4 tumors. Therefore, we subdivided non-WNT/non-SHH tumors into three types: Group 3, Group 4 and Intermediate 3/4 Group.

### SCNAIP expression is lower in the Intermediate 3/4 Group than in Group 4

**Fig. 2:**
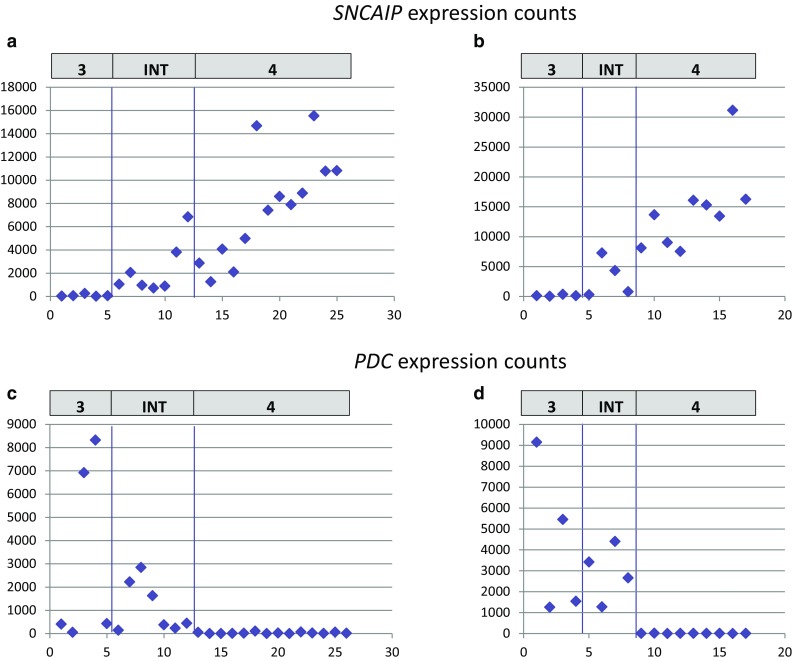
*SNCAIP* and *PDC* expression in three molecular groups. **a, b** *SNCAIP* expression is shown in three molecular groups in a series of 26 and 17 medulloblastomas. Values in the Group 4 are significantly higher than in Intermediate 3/4 Group (p = 0.0013 and p = 0.0005, respectively). **c, d** *PDC* expression is shown in the same set of tumors as in part a and b. Group 4 shows low expression of *PDC* gene as compared to other groups (p = 0.007 and p = 0.0005, respectively). The values are presented as raw NanoString counts after normalization

*SCNAIP* gene duplication and expression has been associated with Group 4 in the previous study [5]. Our analysis performed across two sets of 44 and 24 samples showed that *SCNAIP* was expressed at a significantly lower level in the Intermediate 3/4 Group than in Group 4 in both series (p = 0.0013 and p = 0.0005, respectively), (Fig. 2 a, b). Therefore, tumors from Intermediate 3/4 Group unlikely include duplicated *SCNAIP* cases which are associated with higher expression levels of *SCNAIP* gene.

### PDC expression is a marker for Group 3 and Intermediate 3/4 Group

High expression of *PDC* gene was present in Group 3 and Intermediate 3/4 Group, but showed low expression in all Group 4 tumors (p = 007 and p = 0.0005; Fig. 2 c, d).

**Fig. 3:**
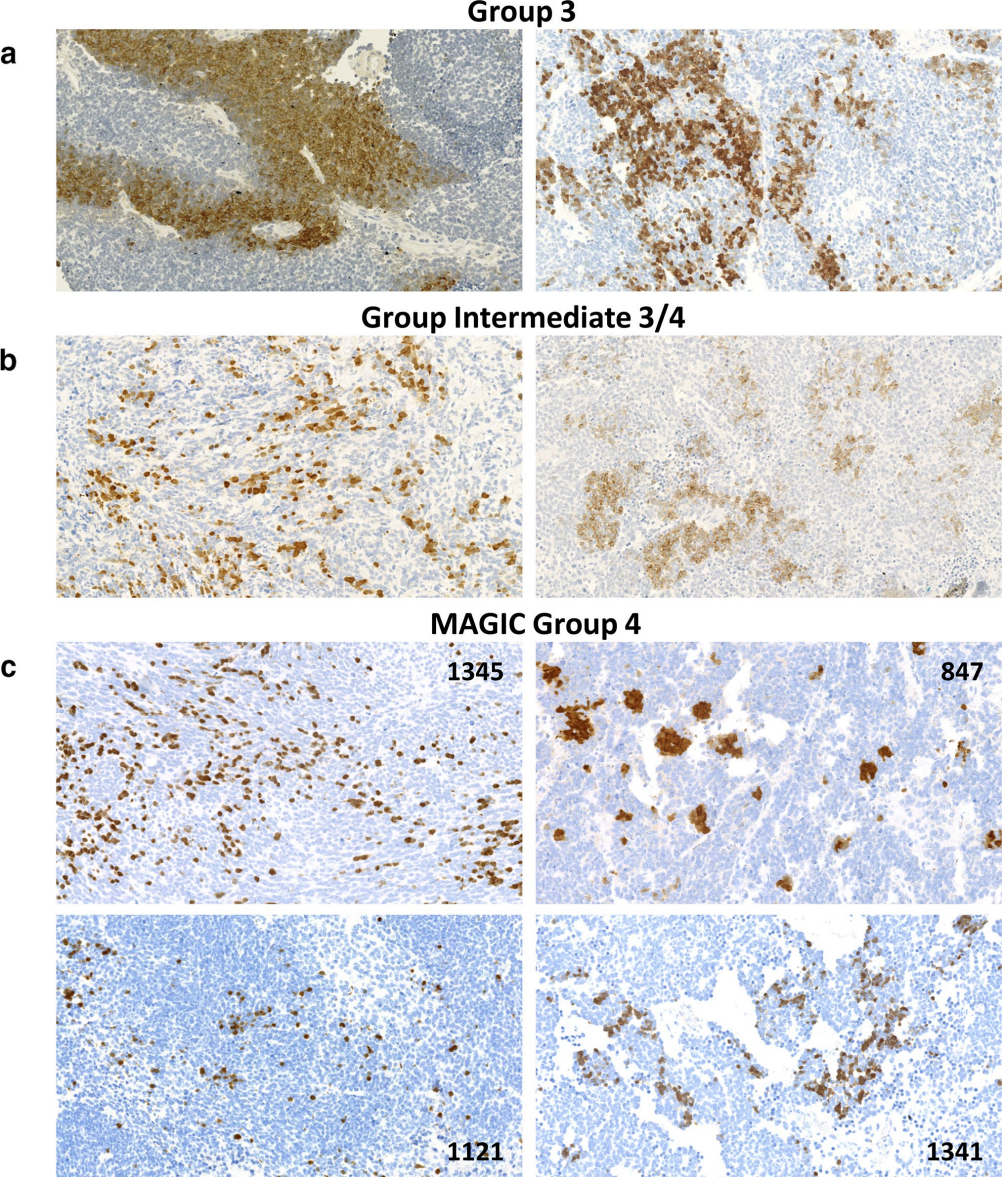
Phosducin (PHOS) expression in medulloblastomas. Examples of positive PHOS expression in Group 3 (**a**), Intermediate 3/4 Group (**b**) and medulloblastomas from the MAGIC study Group 4 (**c**). The number on image represents ID of the patient. Images were scanned at original magnification ×40 and presented digital magnification is ×20

To confirm these results at the PDC protein (PHOS, phosducin) level, we performed immunohistochemical analysis using antibody anti-PHOS (ab77523) in a series of FFPE preparations. Intense immunoreactivity for PHOS, mainly cytoplasmic and membrane, was present in all 8 analyzed tumors from Group 3 and in all 10 analyzed tumors from the Intermediate 3/4 Group (Fig. 3a, b). Single tumors in both groups displayed low *PDC* expression at the RNA level but positive immunohistochemical reaction. By contrast, all 23 analyzed tumors from Group 4 showed a negative reaction with only focal expression in individual scattered cells, whereas large areas of the tumor were completely negative. Occasionally, the positively stained fibres in the background of neoplastic tissue were seen.

We also analyzed additional 16 tumors, which were previously investigated and classified as Group 4 tumors by the Medulloblastoma Advanced Genomics International Consortium (MAGIC) study Northcott et al. [5, 7] and found 5 tumors with positive reaction with anti-PHOS antibody (examples shown in Fig. 3c).

### Further characteristics of three groups from non-WNT/non-SHH tumors

Clinical and genetic features present in the analyzed patients are shown in Table 1. Group 3 was characterized by the lower age of patients and more frequent presence of LCA pathology, as compared with Group 4 (p = 0.028 and p = 0.045, respectively). Although the presence of metastases was more frequent in Group 3 (55.6%) as compared to other groups (36.4% for Intermediate 3/4 Group and 40.9% for Group 4), this difference was not significant. Male to female ratio was 2.9:1 for all non-WNT/non-SHH tumors, without significant difference between three groups.

**Table 1:** Characteristics of medulloblastoma patients with non-WNT/non-SHH tumors

ID	Group	Gender	Age years	Metastases	Histopathology	Patient status	PFS mts	OS mts	iso17 or 17q gain	PHOS IHC
1	INT 3/4	m	5	M0M1	Classic	ADF	69	69	+	+
2	INT 3/4	f	8	M2,3	Classic	ADF	88	88	nd	−
3	INT 3/4	f	7	M0M1	Classic	ADF	67	67	+	+
4	INT 3/4	m	5	M2,3	Classic	ADF	48	48	−	+
5	INT 3/4	f	8	M2,3	Classic	ADF	45	45	+	+
6	INT 3/4	f	7	M2,3	LCA	ADF	89	89	nd	+
7	INT 3/4	f	15	M0M1	LCA	ADF	67	67	nd	+
8	INT 3/4	m	6	M0M1	Classic	ADF	48	48	+	+
9	INT 3/4	m	12	M0M1	Classic	ADF	111	111	nd	+
10	INT 3/4	m	13	M0M1	Classic	ADF	55	55	+	−
11	INT 3/4	m	5	M0M1	MBL	ADF	72	72	nd	nd
12	3	m	3	M2,3	LCA	DOD	14	15	+	+
13	3	f	6	M2,3	LCA	DOD	12	29	+	+
14	3	f	17	M0M1	Classic	DOD	24	26	nd	nd
15	3	m	13	M0M1	LCA	DOD	25	26	+	+
16	3	m	8	M2,3	Classic	DOD	24	44	nd	+
17	3	m	1.5	M2,3	LCA	DOD	26	29	+	+
18	3	m	11	M0M1	LCA	ADF	122	122	nd	+
19	3	m	5	M0M1	Classic	ADF	126	126	+	+
20	3	m	3	M2,3	Classic	ADF	68	68	+	+
21	4	m	10	M2,3	Classic	DOD	12	24	+	−
22	4	m	15	M2,3	NOS	DOD	60	97	nd	−
23	4	m	12	M2,3	Classic	DOD	37	42	+	−
24	4	f	13	M0M1	LCA	Secondary leukaemia	36	36	nd	−
25	4	m	14	M0M1	Classic	DOD	26	32	nd	−
26	4	m	17	M2,3	LCA	DOD	22	23	−	−
27	4	m	10	M0M1	Classic	DOD	35	46	+	−
28	4	m	14	M2,3	Classic	ADF	83	83	+	−
29	4	m	8	M2,3	Classic	ADF	73	73	+	−
30	4	m	9	M0M1	Classic	ADF	57	57	+	−
31	4	m	11	M0M1	Classic	ADF	85	85	+	−
32	4	m	5	M2,3	Classic	ADF	191	191	nd	−
33	4	m	14	M2,3	LCA	ADF	57	57	+	−
34	4	m	8	M2,3	Classic	ADF	142	142	nd	−
35	4	m	10	M0M1	Classic	ADF	113	113	+	−
36	4	m	9	M0M1	Classic	ADF	61	61	−	−
37	4	f	12	M0M1	Classic	ADF	119	119	+	−
38	4	m	14	M0M1	Classic	ADF	51	51	+	−
39	4	m	5	M0M1	Classic	ADF	48	48	nd	−
40	4	m	7	M0M1	Classic	Secondary GBM	101	101	nd	−
41	4	f	15	M0M1	Classic	ADF	61	61	nd	−
42	4	m	10	M0M1	NOS	ADF	73	73	nd	−
43	4	f	4	na	LCA	na	na	na	nd	−
1341	MAGIC 4	m	7	M0M1	Classic	ADF	102	102	+	+
1345	MAGIC 4	f	12	M0M1	Classic	ADF	97	97	+	+
847	MAGIC 4	m	12	M3	LCA	ADF	97	97	−	+
1121	MAGIC 4	m	4	M0M1	Classic	ADF	91	91	−	+
132	MAGIC 4	f	7	M0M1	Classic	ADF	159	159	nd	+

*MAGIC 4* Group 4 according to the Medulloblastoma Advanced Genomics International Consortium study, *LCA* large cell/anaplastic, *NOS* not otherwise specified, *ADF* alive disease free, *GBM* glioblastoma, *PHOS* phosducin, *IHC* immunohistochemistry, *na* not available, *nd* not done, *m* male, *f* female, *mts* months

Two tumors from Group 3 (No 12 and 18) displayed a high level of *MYCC* expression and FISH analysis identified high amplification (> 10 copy number) of *MYCC* gene in those cases.

Isochromosome 17q or 17q gain, as detected by MLPA, was present in 88% of analyzed cases and the frequency was not significantly different between the groups.

### Survival analyses for three groups from non-WNT/non-SHH tumors

**Fig. 4:**
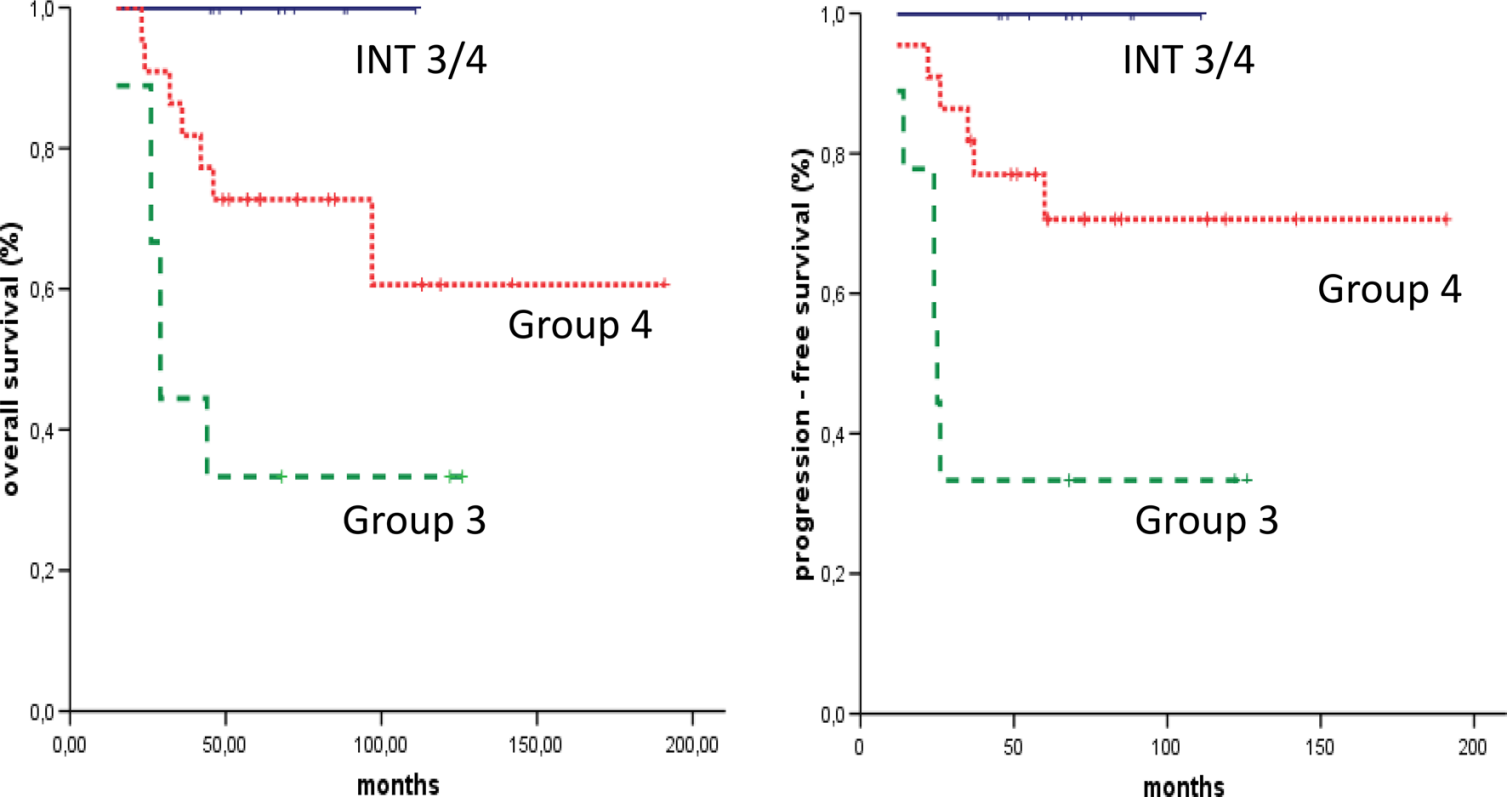
Survival analyses in medulloblastoma according to three molecular groups as detected by NanoString method. Final overall survival values were: 100% for INT3/4 group, 33.3% for Group 3 and 60% for Group 4. Final progression free survival values were: 100% for INT3/4 group, 33.3% for Group 3 and 70.6% for Group 4. *INT3/4* group Intermediate 3/4

5 year OS and PFS were significantly better for Intermediate 3/4 Group (n = 11, 100% both), when compared with Group 3 (n = 9, 33.3% both, p = 0.001) and better when compared to Group 4 (n = 22, 72.7 and 70.6%, p = 0.061 and p = 0.066, respectively), (Fig. 4).

Additionally, we analyzed 5 patients from the MAGIC Group 4, which displayed positive PHOS reaction and therefore may be categorized as an Intermediate 3/4 Group. All 5 patients are the long time survivors. In consequence, for the increased set of tumors OS and PFS were still significantly better for Intermediate 3/4 Group when compared with Group 3 (p = 0.0001, for both OS and PFS) and also significantly better when compared with Group 4 (p = 0.025 and p = 0.023, respectively).

## Discussion

The results from several previous studies clearly identified the WNT and the SHH medulloblastomas, regardless of the method applied. By contrast, the remaining non-WNT/non-SHH tumors warrant further characterization in order to establish clinically more effective sub-grouping of these tumors. Although they are provisionally categorized into Group 3 and Group 4 in the recent WHO 2016 classification [6] it is likely that number of cases do not fit into any of these two groups.

Earlier studies based on expression microarrays using Affymetrix HG U133 Plus 2.0 Array identified 3–4 groups within non-WNT/non-SHH tumors, with several tumors displaying an expression of mixed signatures, e.g. group D in Kool et al. study [1] and group c4 in Cho et al. study [3]. The latter tumors included the presence of distinct subpopulations of CRX or GRM8 immunopositive cells, what may explain their mixed gene expression signature.

By contrast, two groups within non-WNT/non-SHH tumors were recognized by subsequent analyses based on Affymetrix Human Exon 1.0 ST Array and unsupervised hierarchical clustering of 1450 high–standard deviation genes. However, principal component analysis (PCA) showed overlapping of samples within these groups [4]. Also, two groups were revealed in the study based on gene methylation profiling using Illumina HumanMethylation450 BeadChip array and unsupervised *k*-means consensus clustering. Still, when the samples were compared with the gene expression sub-grouping, several incompatible cases involved switches between Group 3 and Group 4, but not between the WNT and SHH groups [12]. Finally, the 22-genes expression classifier based on NanoString technology revealed high concordance with the results obtained using Affymetrix Human Exon 1.0 ST Array when the partitioning around medoids (PAM) algorithm was applied. Then again, when different class prediction algorithms were employed, some samples could not be reliably assigned into defined two groups [7]. Therefore, an uncertainty regarding categorization of some non-WNT/non-SHH tumors still persists.

As the result of an ambiguous classification, proportion of tumors belonging to Group 3 or Group 4 may vary, also in the large analyzed series. For example, ratio of Group 3 tumors to Group 4 tumors was 1:1.35 for exon array data, 1:1.56 for methylation profiling data and 1:2 for NanoString data [4, 5, 12]. This is important, since the presence of ambiguous cases may influence the results of clinical-molecular correlations.

From the clinical point of view, the most important issue is related to the survival rate of patients. Patients with Group 3 tumors showed consistently worse prognosis than patients with Group 4 but the difference varied from significant to not significant, depending on the study [2, 5, 13, 14]. This may result from the number of factors, however, it clearly shows that the non-WNT/non-SHH medulloblastoma should be investigated further to establish meaningful risk group stratification for patients.

In our study, we recognized three transcriptional groups within non-WNT/non-SHH tumors. Although the results were obtained on the limited number of cases and marker genes, the identified Group 3 showed all features of poor prognosis, including frequent metastases, LCA pathology and presence of tumors with *MYCC* amplification. Importantly, survival rate was < 40% and significantly lower than in the remaining groups. This was not contributed to the presence of two *MYCC* amplified cases, since one of the patient has been a long time survivor. Patients with Group 4 medulloblastoma had an intermediate survival rate what is expected from the previous studies. The most noteworthy result is related to the Intermediate 3/4 Group where patients > 3 years of age showed an excellent survival rate. This was the only statistically significant feature which could distinguish this group from the other two in our series. Although we did not provide further extensive molecular characteristics of tumors, it seems that tumors with transitional features, as detected in this study, may constitute a clinically relevant cohort. They may be compatible with group c4 which showed > 90% OS and > 75% EFS rates [3].

Recently, four risk groups based on DNA methylation profiling were proposed for non-WNT/non-SHH tumors where Group 3 and Group 4 were subdivided further into high risk (HR) and low risk (LR) tumors [15]. Interestingly, Group 3 LR and Group 4 LR shared a common metagene (V1) and were considered as a single entity for clinical purposes. It is tempting to speculate that these tumors may be also compatible with our Intermediate 3/4 Group what would further justify a necessity for more than two groups prognostic classifier within non-WNT/non-SHH tumors.

In our study, we applied the NanoString method as a potentially useful diagnostic tool for analysis of FFPE material. The increased number of genes, including two genes regulating phototransduction cascade, allowed for identification of tumors, which shared common Group 3 and Group 4 signatures and the existence of these “middle ground” tumors allowed for the separation of “genuine” Group 3 from Group 4. Expression of *SNCAIP* gene was significantly higher in Group 4 than in the Intermediate 3/4 Group, therefore we assume that our Group 4 tumors may include cases recognized as group 4α by Northcott et al. [5].

From the diagnostic point of view, it seems that the NanoString method requires further exploration in terms of the establishment of the most clinically relevant genes signature and algorithms used for the tissue samples clustering. The fact that several tumors could be originally classified as either Group 3 or Group 4 medulloblastoma in our study, depending on the algorithm applied, is not acceptable for diagnostic purposes. Therefore, we suggest that more effort should be undertaken to adjust nonetheless a very promising NanoString method for a better classification of non-WNT/non-SHH tumors.

Also, since there are currently no reliable immunohistochemical markers for individual groups detection, we suggest that expression of PDC protein present in Group 3 and Intermediate 3/4 Group may be one of the useful biological markers for the future diagnostic panel.

In summary, our work supports the view that within the non-WNT/non-SHH tumors different risk groups exist. We confirmed the presence of the poor prognostic group (Group 3) and proved that also favorable group, conventionally named as Intermediate 3/4 Group, exists. This altogether suggests, that the current two groups classifier is not sufficient for proper clinical categorization of individual patients.
